# Evolutionary Changes in DnaA-Dependent Chromosomal Replication in Cyanobacteria

**DOI:** 10.3389/fmicb.2020.00786

**Published:** 2020-04-28

**Authors:** Ryudo Ohbayashi, Shunsuke Hirooka, Ryo Onuma, Yu Kanesaki, Yuu Hirose, Yusuke Kobayashi, Takayuki Fujiwara, Chikara Furusawa, Shin-ya Miyagishima

**Affiliations:** ^1^Department of Gene Function and Phenomics, National Institute of Genetics, Shizuoka, Japan; ^2^Research Institute of Green Science and Technology, Shizuoka University, Shizuoka, Japan; ^3^Department of Applied Chemistry and Life Science, Toyohashi University of Technology, Toyohashi, Japan; ^4^Department of Genetics, The Graduate University for Advanced Studies (SOKENDAI), Shizuoka, Japan; ^5^Center for Biosystems Dynamics Research, RIKEN, Osaka, Japan; ^6^Universal Biology Institute, Graduate School of Science, The University of Tokyo, Tokyo, Japan

**Keywords:** CDS skew, chromosome replication, cyanobacteria, DnaA, GC skew, polyploidy

## Abstract

Replication of the circular bacterial chromosome is initiated at a unique origin (*oriC*) in a DnaA-dependent manner in which replication proceeds bidirectionally from *oriC* to *ter*. The nucleotide compositions of most bacteria differ between the leading and lagging DNA strands. Thus, the chromosomal DNA sequence typically exhibits an asymmetric GC skew profile. Further, free-living bacteria without genomes encoding *dnaA* were unknown. Thus, a DnaA-*oriC*-dependent replication initiation mechanism may be essential for most bacteria. However, most cyanobacterial genomes exhibit irregular GC skew profiles. We previously found that the *Synechococcus elongatus* chromosome, which exhibits a regular GC skew profile, is replicated in a DnaA-*oriC*-dependent manner, whereas chromosomes of *Synechocystis* sp. PCC 6803 and *Nostoc* sp. PCC 7120, which exhibit an irregular GC skew profile, are replicated from multiple origins in a DnaA-independent manner. Here we investigate the variation in the mechanisms of cyanobacterial chromosome replication. We found that the genomes of certain free-living species do not encode *dnaA* and such species, including *Cyanobacterium aponinum* PCC 10605 and *Geminocystis* sp. NIES-3708, replicate their chromosomes from multiple origins. *Synechococcus* sp. PCC 7002, which is phylogenetically closely related to *dnaA*-lacking free-living species as well as to *dnaA*-encoding but DnaA-*oriC*-independent *Synechocystis* sp. PCC 6803, possesses *dnaA*. In *Synechococcus* sp. PCC 7002, *dnaA* was not essential and its chromosomes were replicated from a unique origin in a DnaA-*oriC* independent manner. Our results also suggest that loss of DnaA-*oriC*-dependency independently occurred multiple times during cyanobacterial evolution and raises a possibility that the loss of *dnaA* or loss of DnaA-*oriC* dependency correlated with an increase in ploidy level.

## Introduction

Precise chromosomal DNA replication is required for the inheritance of genetic information during cellular proliferation. Chromosome replication is tightly controlled, mainly at the initiation stage of DNA replication (Wang and Levin, 2009; Katayama et al., 2010; Skarstad and Katayama, 2013). In eukaryotes and certain archaea, chromosome replication is initiated at multiple origins scattered throughout chromosomes (Bell, 2017; Marks et al., 2017). In contrast, in most bacteria with a single circular chromosome, chromosome replication is initiated at a unique origin named *oriC* (Katayama et al., 2010; Skarstad and Katayama, 2013). Two replication forks assembled at *oriC* proceed bidirectionally around the circular chromosome, simultaneously synthesizing the nascent leading and lagging DNA strands. Replication of circular chromosomal DNA terminates in the *ter* region, located at a site opposite to that of *oriC* (Duggin et al., 2008; Beattie and Reyes-Lamothe, 2015; Dewar and Walter, 2017). *oriC* comprises several copies of the DnaA-box sequence (TTATNCACA) that is bound by DnaA. DnaA unwinds the duplex DNA to form single-stranded DNA templates. Subsequently, the replisome is recruited to the unwound DNA and then initiates DNA synthesis (Katayama et al., 2010). Free-living bacteria, which do not possess *dnaA*, were not reported, and evidence suggested that the DnaA-*oriC*-dependent mechanism of chromosomal DNA replication initiation is conserved among bacteria, except for certain symbionts and parasites, which do not harbor *dnaA* (Akman et al., 2002; Gil et al., 2003; Klasson and Andersson, 2004; Ran et al., 2010; Nakayama et al., 2014).

Nucleotide compositional bias and gene orientation bias between the leading and lagging DNA strands occur in most bacterial chromosomes (Lobry, 1996; Freeman et al., 1998; Bentley and Parkhill, 2004; Nikolaou and Almirantis, 2005; Necşulea and Lobry, 2007). GC skew, defined as (G – C)/(G+C), switches near *oriC* and *ter* (Lobry, 1996; Grigoriev, 1998). Further, coding-sequence orientation bias (CDS skew) switches near *oriC* and *ter* (Freeman et al., 1998; Nikolaou and Almirantis, 2005; Necşulea and Lobry, 2007), because numerous genes, particularly those abundantly expressed or those essential for viability, are encoded on the leading, rather than the lagging strand (Rocha and Danchin, 2003). Moreover, the replication-associated *dnaA-dnaN* operon resides near *oriC* (Mackiewicz et al., 2004). These conserved footprints on the bacterial chromosome and conservation of *dnaA* indicate that the DnaA-*oriC*-dependent mechanism for chromosome replication is highly conserved in bacteria.

The GC and CDS skews and location of the *dnaA-dnaN* operon are used to computationally predict the position of *oriC* in numerous bacterial chromosomal genomes (Luo and Gao, 2018). However, the availability of an increasing number of bacterial genomes, accelerated through the rapid development of nucleotide sequencing technologies, show that the replication origin of certain bacterial genomes cannot be predicted from the genomic sequence. Examples include cyanobacterial genomes with irregular GC and CDS skews (McLean et al., 1998; Nikolaou and Almirantis, 2005; Worning et al., 2006; Arakawa and Tomita, 2007). Further, *dnaA* and *dnaN* are encoded separately in these genomes (Zhou et al., 2011; Huang et al., 2015). In addition, most cyanobacterial species possess multiple copies of the same chromosomes (Simon, 1977; Binder and Chisholm, 1990, 1995; Hu et al., 2007; Zerulla et al., 2016), in contrast to those present in single copies in model bacteria such as *Escherichia coli*, *Bacillus subtilis*, and *Caulobacter crescentus*.

**FIGURE 1:**
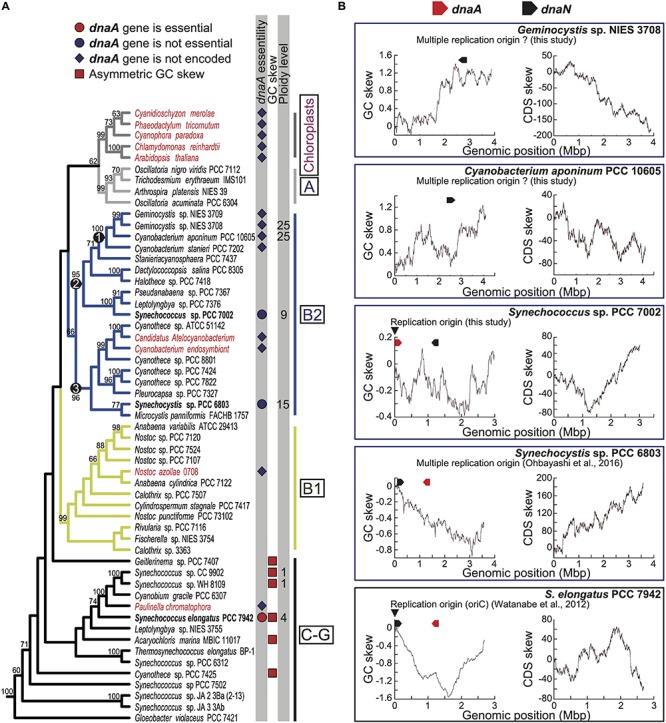
Phylogenetic distributions of *dnaA* among cyanobacterial and chloroplast genomes and GC and CDS skew profiles of chromosomes. **(A)** Phylogenetic relationships of cyanobacteria and chloroplasts and presence or absence of genomic *dnaA*. The tree was constructed using a maximum likelihood method based on 58 concatenated rDNA sequences (16S + 23S + 5S rDNA). Bootstrap values > 50% are shown above the selected branches. The full tree with the outgroup and accession numbers of respective nucleotide sequences are indicated in Supplementary Figure S1. Clades A, B1, B2, and C-G are defined according to a previous study (Shih et al., 2013). Chloroplasts and symbiotic species are shown in red, and free-living species are shown in black. The red or blue circle next to the species name indicates that *dnaA* was experimentally shown as essential or not essential for chromosome replication in the species (Ohbayashi et al., 2016; present study). The blue diamond next to the species name indicates the absence of *dnaA*. The red square next to the species name indicates that the chromosomal genome exhibits a clear asymmetric (V-shaped) GC skew profile. **(B)** Cumulative GC and CDS skew profiles of the chromosome of *Geminocystis* sp. NIES-3708, *Cyanobacterium aponinum* PCC 10605, *Synechococcus* sp. PCC 7002, *Synechocystis* sp. PCC 6803, and *Synechococcus elongatus* (profiles of other species are shown in Supplementary Figures S4, S5). The positions of *dnaA* and *dnaN* are indicated above the profiles. The arrowheads indicate the experimentally determined replication origins of *Synechococcus* sp. PCC 7002 (this study) and *S*. *elongatus* (Watanabe et al., 2012). The chromosome of *Synechocystis* sp. PCC 6803 is replicated from multiple origins (Ohbayashi et al., 2016).

The replication origin was experimentally identified in the chromosome of the model cyanobacterium *Synechococcus elongat*us PCC 7942 (*S*. *elongatus*), which exhibits a regular GC skew (Watanabe et al., 2012). In this genome, *oriC* resides near *dnaN*, which corresponds to one of the two shift points of the GC skew (Watanabe et al., 2012) (Figure 1). Further, DnaA binds *oriC*, and *dnaA* is essential in *S*. *elongatus* (Ohbayashi et al., 2016). In contrast, the chromosomes of *Synechocystis* sp. PCC 6803 and *Nostoc* sp. PCC 7120 exhibit an irregular GC skew profile (Figure 1), and their chromosomes are replicated asynchronously from multiple origins (Ohbayashi et al., 2016). Deletion of *dnaA* does not affect the growth and chromosomal replication activity of these species (Ohbayashi et al., 2016), suggesting that *Synechocystis* sp. PCC 6803 and *Nostoc* sp. PCC 7120 may replicate their chromosomes through an unknown DnaA-*oriC*-independent mechanism (Ohbayashi et al., 2016).

DnaA binds DnaA-boxes in chromosomal regions other than *oriC* to regulate transcription (Messer and Weigel, 1997; Burkholder et al., 2001; Hottes et al., 2005; Ishikawa et al., 2007). Thus, DnaA protein in these cyanobacterial species likely involves the transcriptional regulation of specific genes, but likely is not involved in chromosome replication. Moreover, *dnaA* is not encoded by the genomes of symbiotic cyanobacterial species and chloroplasts, which evolved from a cyanobacterial endosymbiont (Ran et al., 2010; Nakayama et al., 2014; Ohbayashi et al., 2016).

Thus, several species of cyanobacteria, the majority of which possess multiple copies of the same chromosome (Griese et al., 2011; Sargent et al., 2016), likely have developed DnaA-*oriC*-independent mechanism of chromosome replication during evolution, and *dnaA* was subsequently lost from symbionts and chloroplasts. However, the dependence of chromosome replication on DnaA-*oriC* in cyanobacteria other than *S. elongatus, Synechocystis* sp. PCC 6803, and *Nostoc* sp. PCC 7120 and the evolutionary relationships among DnaA-*oriC*-dependent and independent species have not been examined.

To address this gap in our knowledge, here we conducted an up-to-date review of cyanobacterial genome sequences and found that certain free-living species do not encode *dnaA*. *Synechococcus* sp. PCC 7002 is closely related to free-living species without *dnaA* as well as to DnaA-*oriC*-independent *Synechocystis* sp. PCC 6803. We found that the chromosome of *Synechococcus* sp. PCC 7002 is replicated in a DnaA-*oriC* independent manner that occurs in *Synechocystis* sp. PCC 6803, although replication of the chromosome apparently initiated at a specific position. Our results further reveal variations in the mechanism of the initiation of chromosome replication associated with DnaA*-oriC*-dependence and suggest that mechanisms of DnaA-*oriC*-independent replication independently evolved multiple times during cyanobacterial evolution.

## Materials and Methods

### Strains and Culture Condition

*Synechococcus elongatus*, *Synechocystis* sp. PCC 6803, *Cyanobacterium aponinum* PCC 10605, and *Geminocystis* sp. NIES-3708 were cultured in BG-11 liquid medium at 30°C with air bubbling in the light (70 μmol m^–2^ s^–1^ photons). *Synechococcus* sp. PCC 7002 was cultured in modified liquid medium A (Aikawa et al., 2014) with air bubbling at 38°C in the light (70 μmol m^–2^ s^–1^), unless otherwise indicated.

### Construction of GC and CDS Skew Profiles

GC and CDS skew analyses were performed using the G-language Genome Analysis Environment (Arakawa et al., 2003). The cumulative GC and CDS skews were calculated using the “gcskew” and “CDS skew” functions with cumulative parameters, respectively. Accession numbers of the genomic sequences of cyanobacterial species and chloroplasts are indicated in Supplementary Figure S1.

### Phylogenetic Analysis

The concatenated rDNA sequences (16S + 23S + 5S rDNA) of 58 species were automatically aligned using the L-INS-I method of MAFFT v7.299b (Katoh and Standley, 2013). Poorly aligned regions were eliminated using TrimAl v1.2 (Capella-Gutiérrez et al., 2009) with the “-gappyout” option. The aligned sequences were calculated using RAxML v8.2.9 (Stamatakis, 2014) with GTR + GAMMA model, which was selected using Kakusan4 (Tanabe, 2011), and the corresponding bootstrap support values were calculated through ML analysis of 1,000 pseudoreplicates. Accession numbers of the genomic sequences of cyanobacterial species and chloroplasts are indicated in Supplementary Figure S1.

### Chromatin Immunoprecipitation (ChIP) and Qualitative PCR (qPCR) Analyses

ChIP and subsequent qPCR analyses were performed according to Hanaoka and Tanaka (2008) with minor modifications. Cells were fixed with 1% formaldehyde for 15 min at room temperature. The cross-linking reaction was stopped by adding glycine (final concentration, 125 mM) and incubating at room temperature for 5 min. Fixed cells were centrifuged, washed twice with ice-cold Tris-buffered saline (TBS; 20 mM Tris-HCl, pH 7.4, 150 mM NaCl), and stored at −80°C. Fixed cells were disrupted using a Beads Crusher (TAITEC) with glass beads (<106 μm, Sigma-Aldrich) in TBS at 4°C, and the genome DNA was sonicated (Covaris Sonication System, MS Instruments) to generate to approximately 500-bp fragments. After centrifugation for 15 min to sediment the insoluble materials, the supernatant fraction containing genomic DNA was subjected to immunoprecipitation with an anti-HA antibody (clone 16B12, BioLegend), diluted 1:250. Precipitated DNAs were quantified using qPCR with the primer sets oriC-F and oriC-R representing the *oriC* region and 1294-F as well as with 1294-R representing the genomic region farthest from *oriC* in the circular genome. Primer sequences are listed in Supplementary Table S1.

### Flow Cytometric Analysis of DNA and Fluorescence Microscopy of Nucleoids

Exponentially growing cells (Supplementary Figure S2 and Figure 4B) were centrifuged, fixed with 1% glutaraldehyde for 10 min, and washed with PBS. Fixed cells were stained with SYBR Green I (1:3000) and then subjected to flow cytometry (BD, Accuri C6) and observed using fluorescence microscopy (Olympus, BX-52).

### Immunoblotting

Total cellular proteins (80 μg of proteins per lane) were separated using a 10% acrylamide gel and then electrophoretically transferred to a PVDF membrane. Membranes were blocked with 5% skim milk in TBS-T (10 mM Tris-HCl, pH 7.5, 150 mM NaCl, 0.1% Tween 20) and incubated with an anti-HA antibody (clone 16B12, BioLegend) diluted 1:1,000. A horseradish peroxidase–conjugated goat anti-mouse IgG (Thermo Fisher Scientific), diluted 1:20,000, served as the secondary antibody. The immune complexes were detected using ECL Prime Western Blotting Detection Reagent (GE Healthcare) with an Image Quant LAS 4000 Mini (GE Healthcare).

### High-Throughput Nucleotide Sequence Analysis of Chromosome Replication

Genomic DNA was extracted from exponentially growing and stationary phase *S. elongatus*, *C. aponinum* PCC 10605, *Geminocystis* NIES-3708, *Synechococcus* sp. PCC 7002 as well as its *dnaA* disruptant (Supplementary Figure S2). A Covaris S2 Sonication System (Covaris, Inc., Woburn, MA, United States) was used to sonicate DNA samples (5 μg each) to generate approximately 500-bp fragments. Sequencing libraries were prepared using the Ultra II DNA Library Prep Kit for Illumina (New England Biolabs). Paired-end sequencing (320 cycles) was conducted using the MiSeq system (Illumina) according to the manufacturer’s specifications. The sequence reads (number of reads in all samples was >100 per base) were trimmed using CLC Genomics Workbench ver. 8.5.1 (Qiagen) using the parameters as follows: Phred quality score >30, removal of the terminal 15 nucleotides from the 5′ end and 2 nucleotides from the 3′ end, and removal of truncated reads > 100 nucleotides. Trimmed reads were mapped to the reference genome sequences of the respective species using CLC Genomics Workbench ver. 9.5.1 (Qiagen) with the parameters as follows: Length fraction, 0.7 and Similarity fraction, 0.9. To call SNPs and indels, we used the filter settings as follows: minimum read depth for SNP/indel calling = 10, minimum read depth for SNP calling = 5, and 80% cutoff of percentage aligned-reads-calling the SNP per total mapped reads at the SNP sites. Alternatively, potential nucleotide differences were determined using BRESEQ (Deatherage and Barrick, 2014) (Supplementary Data File). Paired-end reads were assembled *de novo* using Newbler version 2.9 (Roche).

### Plasmid Construction and Generation of Stable Transformants

*Synechococcus elongatus* expressing HA-tagged DnaA under the control of the endogenous *dnaA* promoter were generated as follows: PCR was used to amplify *dnaA orf* with the primers 1 and 2; The pNSHA vector (possessing NS I sequences for double-crossover recombination, *trc* promoter, HA-coding sequence, and spectinomycin resistance gene) (Watanabe et al., 2012) was amplified as a linear DNA using PCR with primers 3 and 4. The amplified *dnaA orf* was subsequently inserted between the *trc* promoter and HA-coding sequence of pNSHA using an In-Fusion Cloning Kit (TAKARA). The *dnaA* promoter (300-bp 5′-upstream sequence flanking the *dnaA* translation start codon), which was amplified using PCR with the primers 5 and 6, was cloned immediately upstream of the HA-coding sequences of the vector and then amplified as a linear DNA using PCR with primers 7 and 8 using the In-Fusion Cloning Kit. The resultant vector was used to transform *S. elongatus*.

To generate an *S. elongatus* strain expressing HA-tagged DnaA of *Synechococcus* sp. PCC 7002 or *Synechocystis* sp, *dnaA orf* of *S. elongatus* in the above vector (expressing HA-tagged DnaA of *S. elongatus* driven by the *dnaA* promoter) was replaced with *dnaA orf* of the respective species as follows: The *dnaA orf* of each respective species was amplified using PCR with the primers 9 and 10 for SYNPCC7002_*A0001* or 11 and 12 for *sll0848*, respectively. The PCR product was cloned just downstream of the *dnaA* promoter of *S*. *elongatus* and the HA-coding sequence of the above vector (expressing HA-tagged DnaA of *S. elongatus*), which were amplified as a linear DNA using PCR with the primers 3 and 4 with the In-Fusion HD Cloning Kit. The resultant vector was used to transform *S. elongatus*. The primer sequences are listed in Supplementary Table S1.

To generate an *Synechococcus* sp. PCC 7002 strain expressing SSB-GFP driven by the endogenous *ssb* promoter, the amplicons were prepared as follows: 1. The *ssb orf* (SYNPCC7002_*A0119*) flanked by the 697-bp upstream sequence of *Synechococcus* sp. PCC 7002 was amplified using primers 7 and 8. 2. The *gfp*-(gentamycin resistance gene) *Gm*^r^ fusion was amplified using the primers 9 and 10 from the genomic DNA of *S*. *elongatus ssb-gfp* strain (Ohbayashi et al., 2019) as a template. 3. A 1,000-bp 3′-downstream sequence of *ssb orf* of *Synechococcus* sp. PCC 7002 was amplified using primers 11 and 12. The amplicons were mixed and fused using recombinant PCR with the primers 7 and 12, and the fused product was used to transform cells. Insertion of the *gfp*-*Gm*^r^ into the chromosomal *ssb* locus was confirmed using PCR with primers 7 and 12.

To produce a *dnaA* deletion strain of *Synechococcus* sp. PCC 7002, PCR products were prepared as follows: (1) A 731-bp upstream sequence of *Synechococcus* sp. PCC 7002 *dnaA orf* was amplified using the primers 19 and 20. The kanamycin resistance gene (*Km*^r^) was amplified using primers 21 and 22. (2) A 700-bp 3′-downstream sequence of *Synechococcus* sp. PCC 7002 *dnaA orf* was amplified using primers 23 and 24. Amplified fragments were mixed and fused using recombinant PCR with primers 19 and 24, and the fused product was used to transform cells. Replacement of chromosomal *dnaA* with *Km*^r^ was confirmed using PCR with the primers 19 and 24.

To generate a *Synechococcus* sp. PCC 7002 strain expressing HA-DnaA driven by the endogenous *dnaA* promoter, PCR products were prepared as follows: (1) A 929-bp 5′-upstream sequence of *Synechococcus* sp. PCC 7002 *dnaA orf* was amplified using the primers 25 and 26. (2) An *HA-dnaA* fusion of *Synechococcus* sp. PCC 7002 was amplified using primers 27 and 28 from the vector described above (for expression in *S. elongatus*). (3) *Sp*^r^ was amplified using primers 29 and 30, and a 900-bp 3′-downstream sequence of *Synechococcus* sp. PCC 7002 *dnaA orf* was amplified using primers 31 and 32. Amplified fragments were mixed and fused using recombinant PCR with the primers 25 and 32, and the fused product was used to transform cells. Insertion of sequences encoding *HA* and *Sp*^r^ into the chromosomal *dnaA* locus was confirmed using PCR with primers 25, 32, and 33. The primer sequences are listed in Supplementary Table S1.

To generate a *Synechocystis* sp. PCC 6803 strain expressing HA-DnaA driven by the endogenous *dnaA* promoter, PCR products were prepared as follows: (1) A 700-bp upstream sequence of *Synechocystis* sp. PCC 6803 *dnaA orf* (*sll0848*) was amplified using primers 34 and 45. (2) *HA-dnaA* of *Synechocystis* sp. PCC 6803 fusion was amplified with the primers 36 and 37 from the vector described above (for expression in *S. elongatus*). (3) *Sp*^r^ was amplified using primers 38 and 39. 4. A 700-bp 3′-downstream sequence of *Synechocystis* sp. PCC 6803 *dnaA orf* was amplified using primers 40 and 41. The amplified fragments were mixed and fused using recombinant PCR with the primers 34 and 41, and the fused product was used to transform cells. Insertion of the sequence encoding *HA* and *Sp*^r^ into the chromosomal *dnaA* locus was confirmed using PCR with the primers 33, 34, and 41. The primer sequences are listed in Supplementary Table S1.

## Results

### Distribution of *dnaA* in Cyanobacterial and Chloroplast Genomes

Cyanobacteria emerged on earth more than two billion years ago and globally diversified in numerous, diverse environments (Herrero and Flores, 2008). The genome sequences of 54 cyanobacterial species deposited in the Pasteur Culture Collection were published in Shih et al. (2013), and the complete genome sequences of >500 cyanobacterial species are available in public databases. We first re-examined the distribution of *dnaA* sequences in these completely sequenced cyanobacterial and chloroplast genomes (Figure 1A and Supplementary Figure S1). Consistent with our previous search (Ohbayashi et al., 2016), we did not detect *dnaA* in the genomes of symbiotic cyanobacteria (Figure 1A). The phylogenetic distributions of these symbionts and chloroplasts suggest that loss of *dnaA* independently occurred in their respective ancestors (Figure 1A).

Although it has been believed that *dnaA* is conserved among free-living bacteria (Yoshikawa and Ogasawara, 1991; Messer, 2002; Gao and Zhang, 2007; Katayama et al., 2010), our search revealed that the genomes of the free-living cyanobacterial species *Cyanobacterium stanier*i PCC 7202, *Cyanobacterium* sp. PCC 10605, *Geminocystis* sp. PCC 6308, and *Geminocystis* NIES-3708 and 3709 do not encode *dnaA*. These cyanobacteria formed a monophyletic group (Figure 1A, clade 1). Further, the topology of the phylogenetic tree suggests that the common ancestor of this group (Figure 1A, clade 1) lost *dnaA* independently from the ancestors of the above-mentioned symbiotic species and chloroplasts.

The clade comprising the *dnaA*-negative and *dnaA*-positive free-living species (Figure 1A, clade 2) was further grouped with the clade (Figure 1A, clade 3) that contained *dnaA*-negative endosymbionts and *dnaA*-positive free-living species, grouped as the clade B2 (Figure 1A). Although the data supporting this grouping were not definitive (boot strap value = 66) this conclusion is strongly supported by previous analyses (Shih et al., 2013). Although clade B2 comprised several *dnaA*-positive species as well as *dnaA*-negative species, our previous and present studies showed that, in *Synechocystis* sp. PCC 6803 (Ohbayashi et al., 2016) and *Synechococcus* sp. PCC 7002 (this study, as described later) of this clade, *dnaA* is not essential for genome replication and cell growth. Thus, even in *dnaA*-positive species, chromosomes are replicated in a DnaA-*oriC* independent manner in at least these two species in clade B2.

Consistent with these results, chromosomes in *Synechocystis* sp. PCC 6803 and *Synechococcus* sp. PCC 7002 exhibited irregular GC skew profiles (Figure 1B). In addition, all sequenced species in the clade B2 exhibited irregular GC skew profiles regardless of the presence or absence of *dnaA* (Figure 1B and Supplementary Figure S4) (Typically, the position around the putative DnaA-box sequences near *dnaA* or *dnaN* is defined as a putative replication origin, and the circular genome sequence is deposited as a linear sequence from that position.). This contrasts with clades C-G in which the chromosomes of most species exhibited a clear V-shape (profile with two shift points) except for those of *Synechococcus* sp. PCC 6321 and the cyanobacterium-derived organelle (chromatophore) in *Paulinella chromatophora* (Figure 1B and Supplementary Figure S4). The clades C-G include the model species *S. elongatus* in which the chromosome is replicated from a unique origin (*oriC*), and DnaA is essential for chromosome replication (Watanabe et al., 2012; Ohbayashi et al., 2016).

GC skew profiles in the clade B1 exhibited an intermediate feature between the clades C-G and B2, in which two of the four sequenced genomes exhibited an V-shape profile (Supplementary Figure S4, *Nostoc* sp. PCC 7120 and *Anabaena cylindrica* PCC 7122). GC skew profiles in the clade A and chloroplast did not exhibit V-shape except for that of the chloroplast in *Chlamydomonas reinhardtii*. Overall, the V-shaped GC skew profile apparently tends to be collapsed in the order of clade C-G, B1, B2, to A and chloroplasts (Figure 1 and Supplementary Figure S4). In addition, the ploidy level also apparently tends to increase from the clade C-G to A and chloroplasts although no information on ploidy level was available in the clade B1 (Figure 1A).

Similar to the GC skew, in clade B2, the CDS skew profiles of most species did not exhibit a regular pattern with two shift points (Supplementary Figure S5). However, the exceptions in clade B2 were the chromosomes of *Synechococcus* sp. 7002 and *Stanieria cyanosphaera* PCC 7437, which exhibited CDS skew profiles with a weak V-shape (Figure 1B and Supplementary Figure S5). In *Synechococcus* sp. 7002, the shift points are near positions 0 and 1.2 Mbp, respectively. This observation raised the possibility that chromosomes in certain *dnaA*-positive species in clade B2 are replicated in a DnaA-*oriC-*dependent manner, unlike *Synechocystis* sp. PCC 6803.

To acquire insights into the variation and evolution of the mechanisms of chromosome replication in clade B2, we next examined the manner of chromosome replication in free-living *dnaA*-negative *C. aponinum* PCC 10605 and *Geminocystis* sp. NIES-3708 as well as *dnaA*-positive *Synechococcus* sp. PCC 7002.

### Chromosome Replication in the *dnaA*-Negative Free-Living Species *C. aponinum* PCC 10605 and *Geminocystis* sp. NIES-3708

**FIGURE 2:**
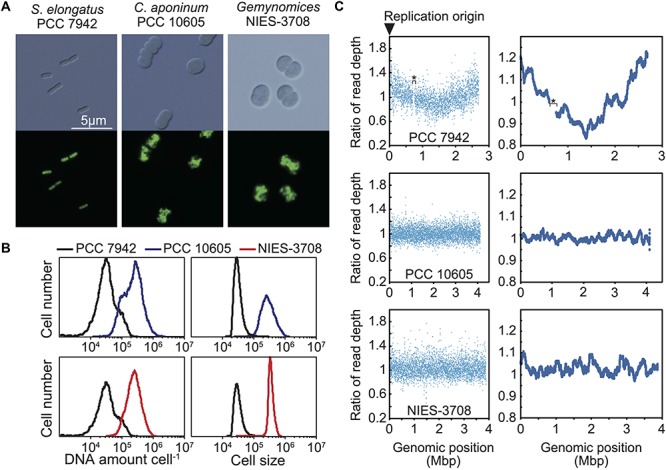
Ploidy and replication manner of the chromosomes of *C. aponinum* PCC 10605 and *Geminocystis* sp. NIES-3708. Exponentially growing *C. aponinum* (chromosome approximately 4.1 Mbp), *Geminocystis* sp. (chromosome approximately 3.9 Mbp), and *S. elongatus* (chromosome approximately 2.7 Mbp; 3–6 copies per cell; Zheng and O’Shea, 2017) (Supplementary Figure S2) were fixed and stained with SYBR Green and then examined using flow cytometry. **(A)** Images of SYBR Green-stained cells. Images were acquired using differential interference contrast microscopy (top) and fluorescence microscopy (bottom). **(B)** Distribution of DNA levels per cell and cell volumes of exponentially growing cultures of *C. aponinum* (blue), *Geminocystis* sp. (red), and *S*. *elongatus* (black). **(C)** Depths of the high-throughput genomic DNA sequence reads at their respective chromosomal regions. Genomic DNA was extracted from the exponentially growing cells (Supplementary Figure S2) and analyzed using an Illumina MiSeq System. Plots of 1-kb (left) and 100-kb windows (right). The number of reads (divided by the number of total reads) of the growing (replicating) cells normalized by that of the stationary phase (non-replicating) cells at each genomic position is shown. The asterisk in the profile of *S. elongatus* indicates a ∼50-kb genomic deletion in our wild type strain which has little effect on replication and cellular growth (Watanabe et al., 2012).

To determine the ploidy of *C. aponinum* and *Geminocystis* sp., exponentially growing cells cultured in an inorganic medium with illumination (Supplementary Figure S2) were stained with the DNA-specific dye SYBR Green I, and the DNA levels and cell sizes were examined using flow cytometry (Figures 2A,B). For comparison, exponentially growing *S. elongatus* cells (3–6 copies of chromosomes per cell) (Chen et al., 2012; Jain et al., 2012; Zheng and O’Shea, 2017) in an inorganic medium with illumination (Supplementary Figure S2) were simultaneously examined (Figures 2A,B). The fluorescence intensity of SYBR Green per *C. aponinum* was approximately 8-times higher compared with that of *S. elongatus* (Figure 2B). The chromosome sizes of *C. aponinum* and *S*. *elongatus* are approximately 4.1 and 2.7 Mbp, respectively. Thus, the result indicates that *C. aponinum* possesses approximately 16–32 copies of its chromosomes. Similarly, the fluorescence intensity of SYBR Green per *Geminocystis* sp. was approximately 8-times higher compared with that of *S. elongatus* (Figure 2B). The chromosome of *Geminocystis* sp. is approximately 3.9 Mbp, indicating that *Geminocystis* sp. possesses approximately 17–34 copies of its chromosomes. Further, the volume of *C. aponinum* and *Geminocystis* sp. cells were approximately 8-times higher compared with that of *S. elongatus* (Figure 2B), showing that an increase in chromosomal ploidy correlated with an increase in cell size.

When bacteria with a single chromosome, such as *Escherichia coli*, grow rapidly in nutrient-rich media, DNA is replicated in a multifork mode, and the *oriC*/*ter* ratio becomes > 2 yielding a V-shaped profile in the depth of high-throughput sequencing reads (lowest at *ter* and highest at *oriC*) (Watanabe et al., 2012). For cyanobacteria possessing multiple copies of the same chromosomes, in which only one or a few copies are replicated from *oriC*, the *oriC*/*ter* ratio approximates 1.0, but still exhibits a V-shaped profile [Watanabe et al., 2012; Figure 2C; The read depth of the exponentially growing (replicating) cells at each genomic potion was normalized by that of stationary phase (non-replicating) cells]. In cyanobacterial species that asynchronously initiate chromosome replication from multiple sites, the ratio of DNA abundance through the genomic position becomes almost constant, as represented by *Synechocystis* sp. PCC 6803 (Ohbayashi et al., 2016). When high-throughput sequence reads of exponentially growing (replicating) *C. aponinum* and *Geminocystis* sp. (Supplementary Figure S2) were mapped to the reference genome, the read depth normalized by that of stationary phase (non-replicating) cells was approximately constant throughout the chromosome (Figure 2C). These results suggest that in the *dnaA*-negative cyanobacteria *C. aponinum* and *Geminocystis* sp., replication of multiple copies of the same chromosomes is asynchronously initiated from multiple sites rather than a unique point, as for *Synechocystis* sp. PCC 6803.

### Dependence of Chromosome Replication on DnaA in *Synechococcus* sp. PCC 7002

**FIGURE 3:**
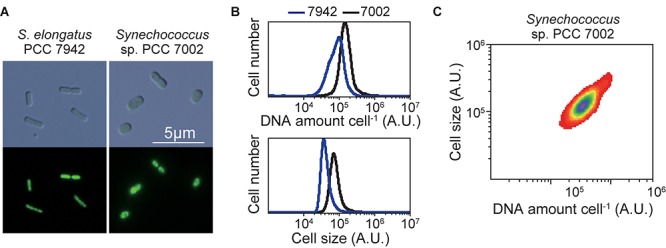
Ploidy of the chromosome of *Synechococcus* sp. PCC 7002. Exponentially growing *Synechococcus* sp. PCC 7002 (chromosome, approximately 3.0 Mbp) and for comparison, *S. elongatus* (chromosome, approximately 2.7 Mbp; 3–6 copies per cell; Zheng and O’Shea, 2017) (Supplementary Figure S2) were fixed and stained with SYBR Green and then examined using flow cytometry. **(A)** SYBR Green-stained cells. Images were acquired using differential interference contrast microscopy (top) and fluorescence microscopy (bottom). **(B)** Distribution of the DNA levels per cell and cell volumes of exponentially growing cultures of *Synechococcus* sp. PCC 7002 (black) and *S*. *elongatus* (blue). **(C)** The relationship between the DNA level and size of *Synechococcus* sp. PCC 7002.

To determine the ploidy of *Synechococcus* sp. PCC 7002, exponentially growing cells in an inorganic medium with illumination (Supplementary Figure S2) were stained with the SYBR Green I, and the DNA level and cell size were examined using flow cytometry (Figure 3). Exponentially growing *S. elongatus* cells (3–6 copies of chromosomes per cell, genome approximately 2.7 Mbp) (Chen et al., 2012; Jain et al., 2012; Zheng and O’Shea, 2017) in an inorganic medium with illumination (Supplementary Figure S2) was simultaneously compared (Figure 3). The fluorescence intensity of SYBR Green I per *Synechococcus* sp. PCC 7002 (genome approximately 3.0 Mbp) was approximately 2-times higher compared with that of *S. elongatus* (Figure 3B). These results indicate that *Synechococcus* sp. PCC 7002 possesses approximately 5–11 copies of its chromosomes, consistent with the findings of others (Moore et al., 2019; this article is preprint). The cell volume of *Synechococcus* sp. PCC 7002 was approximately 2-times higher compare with that of *S*. *elongatus* (Figure 3B). Further, the amount of DNA (chromosome copy number) exhibited a linear, positive correlation with cell volume (Figure 3C).

**FIGURE 4:**
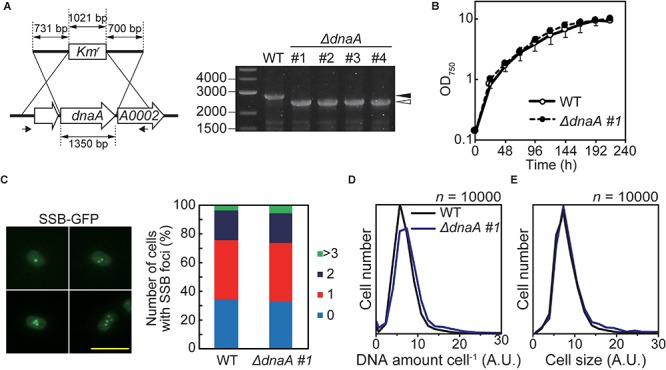
Effect of deleting *dnaA* on the growth and DNA replication of *Synechococcus* sp. PCC 7002. **(A)** Chromosomal *dnaA* was replaced with the gene encoding kanamycin resistance (*Km*^r^) through homologous recombination. Insertion of *Km*^r^ into the chromosomal *dnaA* locus was confirmed using PCR with the primers indicated by the arrows. The wild type (WT) was used as a negative control. The black and white arrowheads indicate the bands amplified from the WT and mutated chromosomes, respectively. The four independent colonies of the *dnaA*-deficient mutant were analyzed. **(B)** Growth of WT and Δ*dnaA* clone #1 in an inorganic medium with illumination (70 μmol m^–2^ s^–1^). Other Δ*dnaA* clones (#2–#4) are shown in Supplementary Figure S6B. **(C)** Frequencies of cells exhibiting zero (blue), one (red), two (deep blue), or >3 (green) SSB-GFP foci in WT and Δ*dnaA* clone #1 cultures 12 h after inoculation (*n* > 300 cells, each strain). For reference, a representative fluorescence image of WT expressing SSB-GFP (green fluorescence) is shown (one to four SSB-GFP foci are shown). Scale bar = 5 μm. The construction of SSB-GFP expresser is described in Supplementary Figure S3. **(D,E)** Flow cytometric analysis showing the distributions of the DNA level per cell **(D)** and the cell volumes **(E)** of cultures of the WT (black line) and Δ*dnaA* clone #1 (blue line) 12 h after inoculation, respectively. DNA was stained with SYTOX Green, and the levels were determined according to the intensity of the green fluorescence.

To examine the dependence of DNA replication on DnaA in *Synechococcus* sp. PCC 7002, we constructed a *dnaA* deletion mutant in which *dnaA* was replaced with a kanamycin resistance gene (*Km*^r^) through homologous recombination. The insertion of *Km*^r^ into the *dnaA* locus was confirmed using PCR and we obtained some transformed clones, in which *dnaA* was completely deleted (Figure 4A). The growth rates of *ΔdnaA* clones were similar to that of wild type (Figure 4B and Supplementary Figure S6B). There was no significant difference in the frequency of chromosome replication between wild type and the *ΔdnaA* mutant (Figure 4C and Supplementary Figure S6C), as indicated by the number of SSB-GFP foci in an exponentially growing cell (Figure 4B and Supplementary Figure S3A). Note that SSB localizes to replication forks (Chen et al., 2012; Mangiameli et al., 2017). Further, the chromosome copy number and cell size of completely segregated *ΔdnaA* clones of *Synechococcus* sp. PCC 7002 were similar to those of the wild type (Figures 4D,E). Thus, there were no significant differences in the chromosome replication and proliferation rates between the wild type and the completely segregated *ΔdnaA*.

**FIGURE 5:**
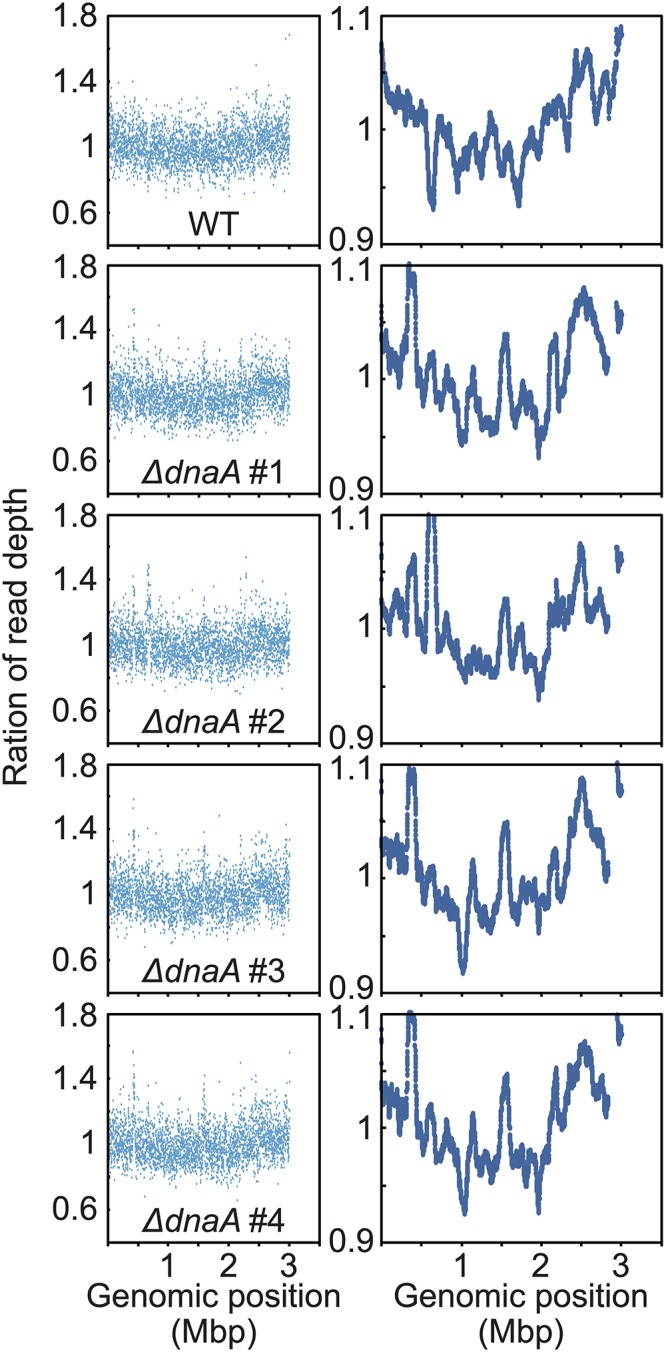
Chromosomal replication origins of *Synechococcus* sp. PCC 7002 WT and *dnaA* disruptants. Depth of the high-throughput genomic DNA reads at respective chromosomal regions in exponentially growing WT and Δ*dnaA* cells. Genomic DNA was extracted from cells 24 h after inoculation (Figure 4B) and analyzed using an Illumina MiSeq System. The number of reads (divided by the number of total reads) of the growing (replicating) cells was normalized by that of the stationary phase (non-replicating) cells at each genomic position. 1-kb window (left) and 100-kb window (right) of WT and Δ*dnaA* clones.

We previously isolated mutants of *Synechocystis* sp. PCC 6803 and *Nostoc* sp. PCC 7120 with completely segregated *ΔdnaA* (Ohbayashi et al., 2016), in which chromosomes were replicated from multiple origins in wild type and *ΔdnaA* cells. Further, complete deletion of *dnaA* does not significantly affect the chromosome replication and proliferation rates of these species (Ohbayashi et al., 2016). In contrast, we isolated completely and incompletely segregated *ΔdnaA* clones of *S*. *elongatus* (Ohbayashi et al., 2016). In these mutants, *ΔdnaA* completely segregated via an episomal plasmid that integrated into the chromosome (Ohbayashi et al., 2016). Further, the replication initiation site of the chromosome shifted from *oriC* to the origin of the integrated plasmid (Ohbayashi et al., 2016). Accordingly, we next determined whether the *ΔdnaA* of *Synechococcus* sp. PCC 7002 harbored an additional suppressor mutation such as that found in *S. elongatus ΔdnaA*. We therefore analyzed the complete genome sequence and profile of genome replication of this mutant (Figure 5 and Supplementary Figure S6).

When the high-throughput sequence reads of exponentially growing wild type *Synechococcus* 7002 (Supplementary Figure S2) were mapped to the reference genome, a V-shaped profile was observed (Figure 5; the read depth of growing cells at each genomic position was normalized that of stationary phase cells). The peak was detected around *dnaA* at position 1 (the leftmost base) (Figure 5), suggesting that the wild type possessed a unique replication origin near *dnaA*. When the sequence reads of the *ΔdnaA* clones harvested during log phase were mapped to the reference genome, all clones exhibited a V-shaped profile similar to that of the wild type (Figure 5), suggesting that the replication of *ΔdnaA* started from a nearby site. Further, mutations such as in-del or point mutation, plasmid integration and transposition of *dnaA* gene were not detected in the *ΔdnaA* clones by high-throughput genomic DNA sequencing (Supplementary Figure S6A and Supplementary Data Sheet). We concluded therefore that *dnaA* was not essential for the proliferation of *Synechococcus* sp. PCC 7002 and that the wild type chromosome replicated in a DnaA-*oriC* independent manner starting from a specific position, unlike that of *Synechocystis* sp. PCC 6803.

### Comparison of the Function and Expression Levels of DnaA in *S. elongatus*, *Synechocystis* sp. PCC 6803, and *Synechococcus* sp. PCC 7002

*Synechococcus* sp. PCC 7002 is phylogenetically closely related to *Synechocystis* sp. PCC 6803 (Figure 1A), in which the chromosome is replicated from multiple origins and *dnaA* is dispensable without additional suppressor mutations (Ohbayashi et al., 2016). Further, the amino acid sequence of DnaA of *S*. *elongatus* is similar to that of *Synechococcus* sp. PCC 7002 (61% identical) and *Synechocystis* sp. PCC 6803 (59% identical). However, previous and the present results suggest that *dnaA* is non-essential for *Synechococcus* sp. PCC 7002 and *Synechocystis* sp. PCC 6803 but is essential for chromosome replication of *S*. *elongatus*.

**FIGURE 6:**
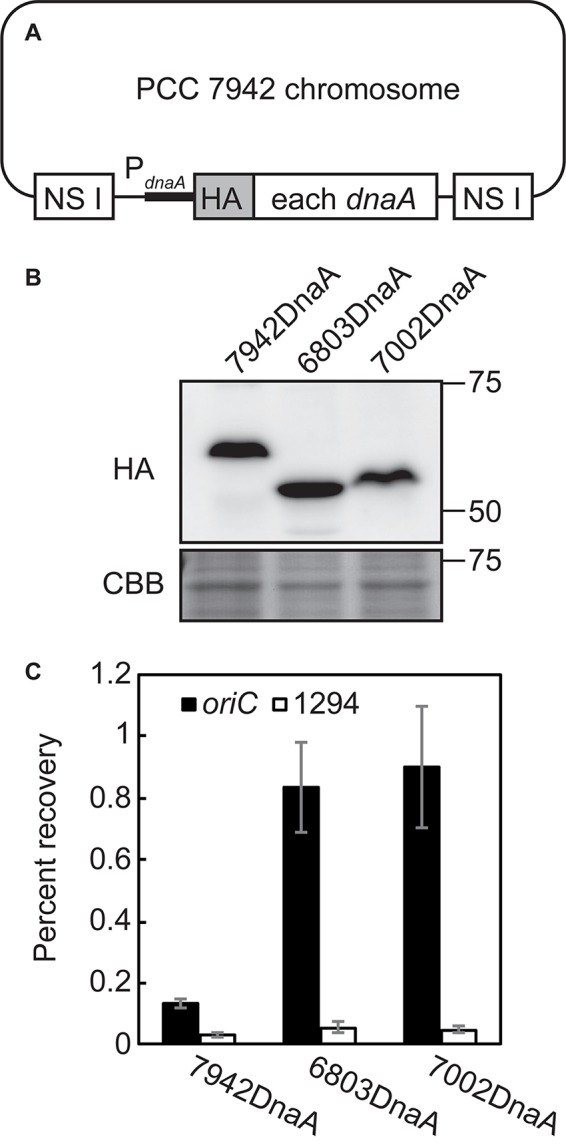
Binding of DnaA to the DnaA-boxes of *Synechococcus elongatus*, *Synechocystis* sp. PCC 6803, and *Synechococcus* sp. PCC 7002. **(A)** Diagram of the *S. elongatus* chromosomes expressing HA-tagged DnaA of *S*. *elongatus* (7942 DnaA), *Synechocystis* (6803 DnaA), and *Synechococcus* 7002 (7002 DnaA). DNA encoding the respective DnaA was integrated into the chromosomal neutral site I (NS I) of *Synechococcus elongatus.* HA-DnaA expression was driven by the promoter of *S*. *elongatus dnaA*. **(B)** Immunoblot analysis of the expression of HA-DnaA in *S*. *elongatus*. Total proteins were subjected to immunoblotting using an anti-HA antibody. Coomassie Brilliant Blue (CBB)-stained proteins are shown as a loading control. **(C)** ChIP-qPCR analysis of the affinity of the binding of DnaA to the *oriC* region (DnaA-boxes) of the *S*. *elongatus* chromosome. The DnaA-chromatin complex was immunoprecipitated using an anti-HA antibody. The samples were quantified using qPCR with the primers representing the *oriC* region (*oriC*: solid bar) and *Syf1294*, which is farthest from *oriC* in the circular chromosome and lacks a DnaA-box sequence (1294: open bar). The percent recoveries of input DNA are indicated with the standard deviation (*n* = 3 biological replicates).

To determine the basis for this difference, we asked whether DnaA molecules in the respective species bind DnaA-box sequences. We therefore expressed HA-tagged DnaAs of *Synechocystis* sp. PCC 6803, *Synechococcus* sp. PCC 7002, and *S*. *elongatus* (positive control) under the control of the *S*. *elongatus dnaA* promoter in *S*. *elongatus* (Figures 6A,B). ChIP-qPCR analysis using an anti-HA antibody revealed that DnaA of each of the three species specifically bound *oriC*, but not *orf*1294, which is farthest from *oriC* in the circular genome and does not possess a DnaA-box sequence (Figure 6C).

**FIGURE 7:**
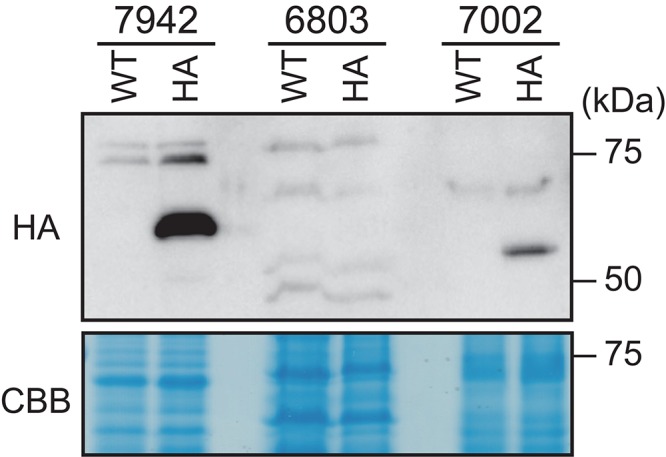
Comparison of DnaA expression levels in *Synechococcus elongatus*, *Synechocystis* sp. PCC 6803, and *Synechococcus* sp. PCC 7002. DNAs encoding HA-tagged DnaA of *S*. *elongatus* (7942), *Synechocystis* sp. PCC 6803 (6803), or *Synechococcus* sp. PCC 7002 (7002) were integrated into the respective chromosomal *dnaA* locus (Ohbayashi et al., 2019; Supplementary Figure S3B,C). HA-DnaA expression was driven by the endogenous *dnaA* promoter. Exponentially growing transformants were inoculated into fresh inorganic medium and cultured for 6 h with illumination (70 μmol m^– 2^ s^– 1^). The same amounts (80 μg) of proteins extracted from the respective cultures were subjected to immunoblotting using an anti-HA antibody. The respective WTs served as negative controls. CBB-stained protein samples are shown as a loading control.

We next expressed HA-DnaA in each of the above species under the control of their respective endogenous *dnaA* promoters (Supplementary Figure S3B,C). Immunoblotting using an anti-HA antibody showed that the level of HA-DnaA in *Synechococcus* sp. PCC 7002 (deduced size including the HA-tag = 54.4 kDa) was lower compared with that of *S*. *elongatus* (deduced size including HA-tag = 55.9 kDa) (Figure 7). Further, we did not detect HA-DnaA in *Synechocystis* sp. PCC 6803 (deduced size including HA-tag = 53.9 kDa) (Figure 7). Thus, in contrast to *S*. *elongatus*, in which *dnaA* is essential for chromosome replication, the level of DnaA was below the detection limit in *Synechocystis* sp. PCC 6803 or very low in *Synechococcus* sp. PCC 7002, in which complete deletion of *dnaA* had no effect on chromosome replication.

## Discussion

The chromosome of *S. elongatus* is replicated from a unique origin (*oriC*) in a DnaA-dependent manner, similar to the mechanism employed by most bacterial species (Ohbayashi et al., 2016). In contrast, in *Synechocystis* sp. PCC 6803 and *Nostoc* sp. 7120, DnaA is not required for chromosome replication, which is initiated from multiple sites (Ohbayashi et al., 2016). Here we extended these findings to show that chromosome replication in *Synechococcus* sp. PCC 7002, which is evolutionarily related to *Synechocystis* sp. PCC 6803, is initiated from a unique origin in a DnaA-independent manner, unlike *Synechocystis* sp. PCC 6803. We further found that certain free-living cyanobacterial species do not possess *dnaA*. The DnaA-*oriC*-independent species *Synechococcus* sp. PCC 7002 and *Synechocystis* sp. 6803, four *dnaA*-negative free-living species, and two *dnaA*-negative endosymbiotic species are phylogenetically closely related (clade B2 in Figure 1A). Their phylogenetic positions suggest that (1) loss of *dnaA* from the common ancestor of the four *dnaA*-negative free-living species (Figure 1A, clade 1), (2) the loss of *dnaA* from the symbiont *Epithemia turgida*, and (3) loss of *dnaA* in the symbiont *Atelocyanobacterium* were independent events. Further, the loss of *dnaA* from other endosymbiotic species (*Nostoc azollae* 0708 and *Paulinella chromatophora*), as well as from the common ancestor of chloroplasts, occurred independently. Similarly, an ancestor of *Nostoc* sp. 7120 (clade B1 in Figure 1A) independently lost DnaA-*oriC*-dependence of an ancestor(s) of *Synechocystis* sp. PCC 6803 and *Synechococcus* sp. PCC 7002 (Figure 1A). Thus, the DnaA*-oriC*-dependent chromosome replication mechanism was lost multiple times during cyanobacterial evolution.

In bacterial groups other than cyanobacteria, there has been no report on free-living species that does not possess *dnaA* gene. However, the genomes of certain bacterial symbionts of insects such as *Wigglesworthia glossinidia* (Akman et al., 2002), *Blochmannia floridanus* (Gil et al., 2003), and Candidatus *Carsonella ruddii* (Nakabachi et al., 2006) do not encode *dnaA*, although they encode genes required for replication (e.g., DNA helicase, DNA polymerase, and DNA primase) (Klasson and Andersson, 2004). Further, mitochondrial and their eukaryotic host genomes do not encode *dnaA* (Ohbayashi et al., 2016). Moreover, certain *dnaA*-negative symbionts possess multiple copies of the same chromosomes as do mitochondria, chloroplasts, and most cyanobacterial species (Bendich, 1987; Griese et al., 2011; Sargent et al., 2016). Thus, loss of DnaA-dependent chromosome replication and loss of *dnaA* are associated with an increase in chromosomal copy number per cell/organelle. At this point, it is unclear how the loss of *dnaA* and endosymbioses are related. One possibility is that loss of a DnaA-*oriC*-regulated mechanism of chromosome replication in endosymbionts was presumably advantageous for host cells to regulate the proliferation of endosymbionts and the replication of their chromosomes.

Another important question is the nature of the function of DnaA in *dnaA*-positive but DnaA-*oriC*-independent species such as *Synechococcus* sp. PCC 7002, *Synechocystis* sp. PCC 6803, and *Nostoc* sp. PCC 7120. Another function of DnaA is to regulate the transcription of a discrete set of genes. For example, *B. subtilis* DnaA binds DnaA-boxes of eight intergenic chromosomal regions and positively or negatively regulates transcription of specific genes (Ishikawa et al., 2007). However, complete disruption of *dnaA* of *Synechocystis* sp. PCC 6803, *Nostoc* sp. PCC 7120 (Ohbayashi et al., 2016), and *Synechococcus* sp. PCC 7002 (this study) did not affect growth under optimal growth conditions. Further, we show here that when *Synechocystis* sp. PCC 6803 is grown under optimal growth conditions, the level of DnaA was below the detection limit. Thus, even if DnaA is involved in transcriptional regulation, the function is not essential in these three DnaA*-oriC*-independent species. At this point, the function of DnaA in these species remains unclear.

Although the GC and CDS skews of most bacteria exhibit clear asymmetric profiles with shift points at *ori* and *ter*, the chromosomes of most cyanobacterial species exhibit irregular patterns. Exceptions such as *S. elongatus*, which exhibits regular GC and CDS skew profiles, were found only in the clades C-G in the phylogenetic tree (Figure 1 and Supplementary Figures S4, S5). The common feature of this group is its relatively lower ploidy level compared with other clades (Griese et al., 2011; Sargent et al., 2016). Further, species with relatively reduced genome sizes only occur in the clades C-G (Shih et al., 2013). Thus, loss of regular GC and CDS skews during cyanobacterial evolution presumably correlated with an increase in the chromosomal copy number, although further characterization of genome copy number in many more cyanobacterial species (species in which ploidy level has not been determined in Figure 1A) is required. It is assumed that in most bacteria the asymmetrical replication machinery between that of the leading strand and that of the discontinuous replication in the lagging strand contributes to differential mutational bias (Bhagwat et al., 2016). Further, evidence indicates that transcriptional mutations create strand-specific nucleotide compositional skew (asymmetric GC skew) (Francino et al., 1996; Rocha et al., 2006; Chen et al., 2016). In most bacteria, genes are preferentially encoded by the leading strand (asymmetric CDS skew), which may be advantageous to avoid the head-on collision of DNA and RNA polymerases (Merrikh et al., 2012; Hamperl and Cimprich, 2016). Further, a preference in the third codon position for G vs. C and T vs. A in bacterial genes may have contributed to the creation of strand-specific nucleotide compositions (Kerr et al., 1997; McLean et al., 1998). In most cyanobacteria, multiple copies of the same chromosomes are replicated asynchronously while transcription occurs in all copies (Ohbayashi et al., 2019). This characteristic of multiple copies of the same chromosomes theoretically reduces the frequency of the head-on collision of DNA and RNA polymerases, leading to loss of regular GC and CDS skews during cyanobacterial evolution.

## Data Availability Statement

All datasets generated for this study are included in the article/Supplementary Material.

## Author Contributions

ROh and SM designed the study. ROh, SH, YKa, and YH performed the nucleotide sequence analyses. ROh and ROn performed the phylogenetic analysis. ROh performed the all other experiments. YKo, TF, and CF provided new reagents and analytical tools. ROh and SM wrote the manuscript.

## Conflict of Interest

The authors declare that the research was conducted in the absence of any commercial or financial relationships that could be construed as a potential conflict of interest.

## Supplementary Material

FIGURE S1 The full phylogenetic tree (partially shown in Figure 1A) with the outgroups and accession numbers of sequences. Branch lengths are proportional to the number of nucleotide substitutions indicated below the tree. Sequences of *Escherichia coli* K-12, *Rhodobacter sphaeroides*, and *Bacillus subtilis* 168 served as an outgroup. The accession numbers are indicated next to the species name. Other details are described in Figure 1.

FIGURE S2 Growth curves of *S. elongatus*, *C. aponinum* PCC 10605, and *Geminocystis* sp. NIES-3708. **(A)** An exponentially growing culture of each species was inoculated into fresh inorganic medium and cultured with air bubbling in the light (70 μmol m^–2^ s^–1^) at 30°C. The arrow indicates the sampling time for the other analyses. The black and white arrowheads indicate sampling points of exponential and stationary phases, respectively. **(B)** Growth rate at the exponential phase.

FIGURE S3 Generation of transformants. **(A)** To express SSB-GFP in *Synechococcus* sp. PCC 7002, the *gfp orf* was integrated into the chromosome immediately before the stop codon of the *ssb* loci of the wild type (WT) and Δ*dnaA* genomes. *Gm*^r^ was used as a selectable marker. Insertion of *gfp* and *Gm*^r^ into the chromosomal *ssb* locus was confirmed using PCR with the primers indicated by the arrows below the illustration. The WT served as a negative control. **(B,C)** To express HA-DnaA under the control of the *dnaA* promoters of *Synechococcus* sp. PCC 7002 **(B)** and *Synechocystis* sp. PCC 6803 **(C)**, DNA encoding HA-DnaA was integrated into the chromosomal *dnaA* locus of each species. *Sp*^r^ was used as a selectable marker. Insertion of the gene encoding *HA* and *Sp*^r^ into the chromosomal *dnaA* locus of each species was confirmed using the primers indicated by the arrows below the illustrations.

FIGURE S4 Cumulative GC skew profiles of cyanobacterial species not shown in Figure 1B. *Nostoc azollae* possesses a pseudo-*dnaA* gene, which is disrupted by insertion of a transposon. Other details are described in Figure 1.

FIGURE S5 Cumulative CDS skew profiles of cyanobacterial species not shown in Figure 1B. The details are described in Figure 1.

FIGURE S6 Growth and chromosomal replication of *Synechococcus* sp. PCC 7002 Δ*dnaA* strains. **(A)** High-throughput genomic DNA reads of WT and Δ*dnaA* strains analyzed using IGV software. Genomic positions (1–3000 bases) including *dnaA* are shown. **(B)** Growth of Δ*dnaA* clones not shown in Figure 4. **(C)** Frequency of cells exhibiting 0 (blue), 1 (red), 2 (deep blue), or ≥3 (green) SSB-GFP foci in Δ*dnaA* clones not shown in Figure 4.

TABLE S1 Primers used in this study.

DATA SHEET S1 BRESEQ analysis of WT *Synechococcus* sp. PCC 7002 and Δ*dnaA* strains.
