# Supplementary figures and images for: Evolutionary Changes in DnaA-Dependent Chromosomal Replication in Cyanobacteria
Source: Front Microbiol. 2020 Apr 28;11:786. doi: 10.3389/fmicb.2020.00786 (PMC7198777; doi:10.3389/fmicb.2020.00786)

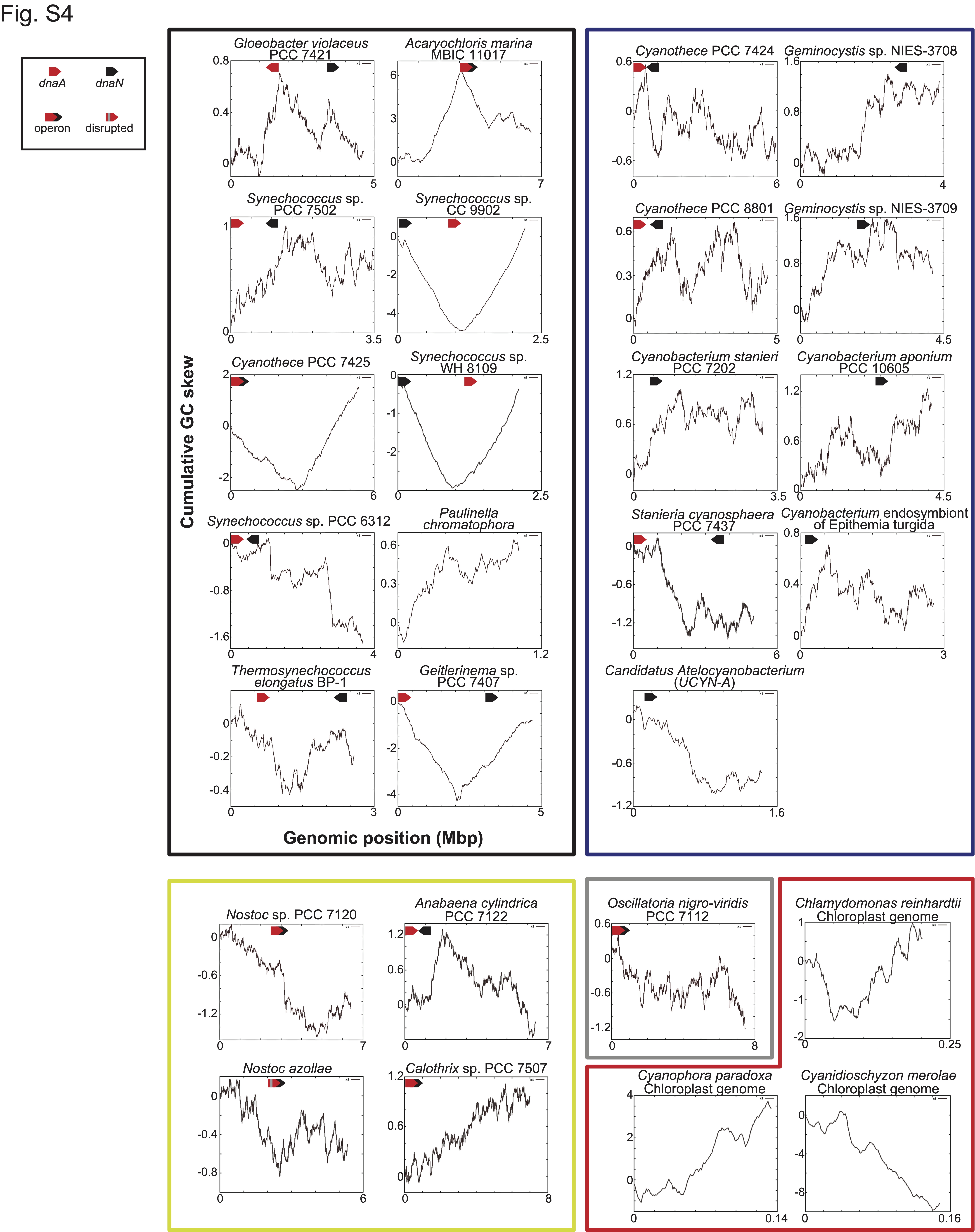

Supplement: FIGURE S4 — Cumulative GC skew profiles of cyanobacterial species not shown in Figure 1B. Nostoc azollae possesses a pseudo-dnaA gene, which is disrupted by insertion of a transposon. Other details are described in Figure 1. [file Image_4.jpg]

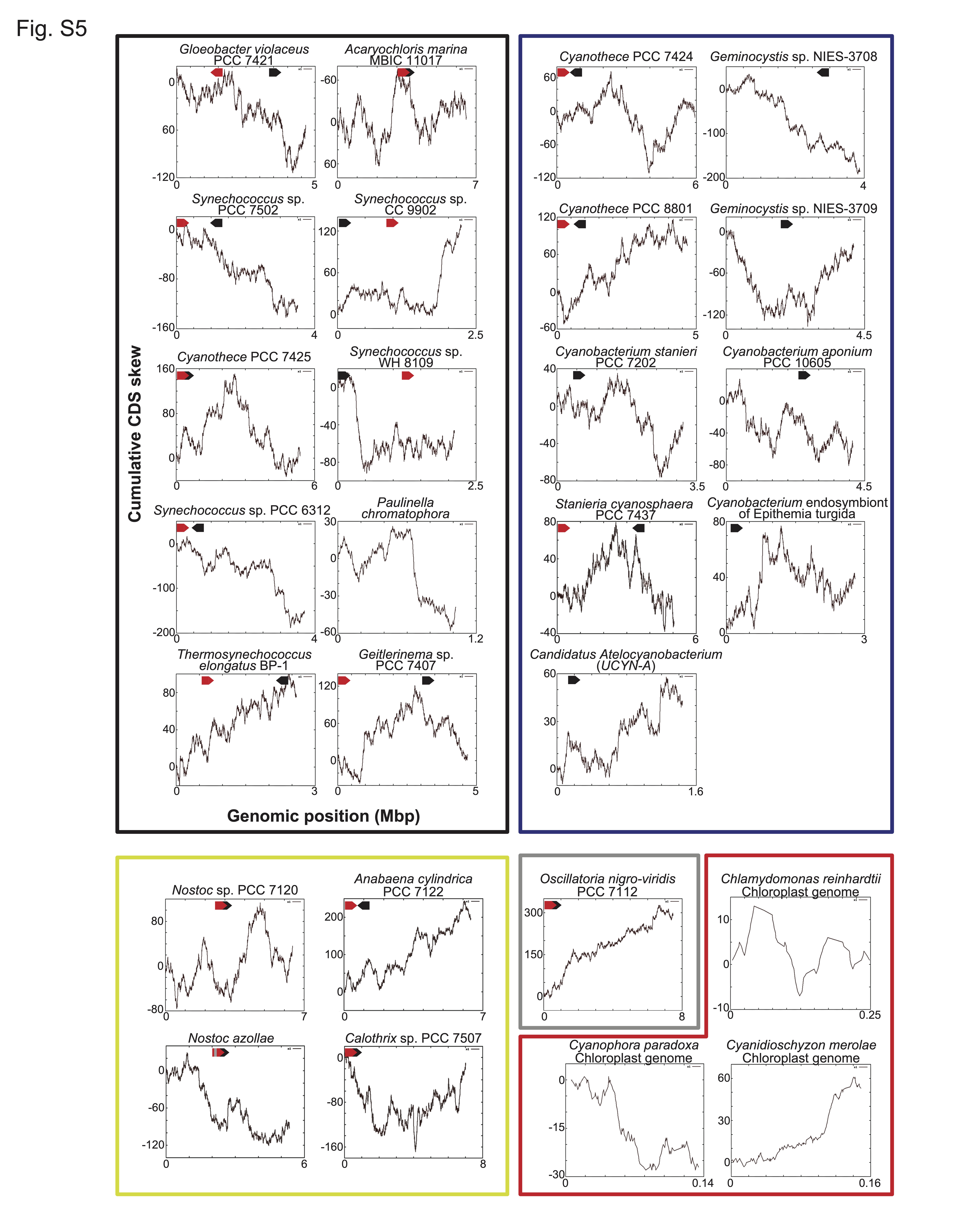

Supplement: FIGURE S5 — Cumulative CDS skew profiles of cyanobacterial species not shown in Figure 1B. The details are described in Figure 1. [file Image_5.jpg]
