# Supplementary material for: Evolutionary Changes in DnaA-Dependent Chromosomal Replication in Cyanobacteria
Source: Front Microbiol. 2020 Apr 28;11:786. doi: 10.3389/fmicb.2020.00786 (PMC7198777; doi:10.3389/fmicb.2020.00786)
Supplement: TABLE S1 — Primers used in this study. [file Table_1.pdf]

Table S1		
Number	Primer name	Seqence 5' --> 3'
1	7942dnaA-F	GACTATGCAGGATCCGTGGAACAGTCCCTAGAAC
2	7942dnaA-R	TGCCTGCAGGTCGACCTAACCGCGGCTAGCTAC
3	HA-F	GTCGACCTGCAGGCATGC
4	HA-R	GGATCCTGCATAGTCCGGGAC
5	7942dnaAus300F	GTTACCACCGCTGCGGCTGCTTCGCTGCGATTT
6	7942dnaAus-R	GAGCTCGAATTCCATAGCGATCGCAGCTGAATAG
7	Ptrd-ds-F	ATGGAATTCGAGCTCCACCGC
8	Ptrc-up-R	CGCAGCGGTGGTAACGGC
9	7002dnaA-F	GACTATGCAGGATCCGTGACTCAAAATCCCCAATG
10	7002dnaA-R	TGCCTGCAGGTCGACCTAGAGGTTTTGGTGATGG
11	6803dnaA-F	GACTATGCAGGATCCATGGTCTCCTGCGAAAATC
12	6803dnaA-R	TGCCTGCAGGTCGACCTAGGATTCCGGGGCTTG
13	7002ssbUS-F	CCACGGAAAAATCTGCCAAAGTTGG
14	7002ssb-R	TACTGTCGACCCCGTAGTTGTTATAATTATCTACCGC
15	GFP-F	CAACTACGGGGTCGACAGTAAAGGAGAAGAACTTTTCAC
16	Gm-R	CGGAGGGTGTTTAGGTGGCGGTACTTGGGTCG
17	7002ssbDS-F	CGCCACCTAAACACCCTCCGCCTGCGGC
18	7002ssbDS-R	CAGCCGGATTTGAATTTACTGGCAG
19	7002rpoC2-F	CCGGATCGAAGAACTCCTCGAAG
20	7002rpoC-R	TACTGTCGACTTGGTCTTTCATCCAATTATCATCAATC
21	GFP-F	GAAAGACCAAGTCGACAGTAAAGGAGAAGAACTTTTCAC
22	Spec-R	TAGATTGCCATTATTTGCCGACTACCTTGGTGATC
23	7002rpoC2DS-F	CGGCAAATAATGGCAATCTAATCGCCAAAAATCATC
24	7002rpoC2DS-R	CGATGTCGCCAAGGAATCCTTG
25	7002dnaAus-F	CGTAAGGACGCTCGCGCC
26	7002dnaAus-R	CAATTCCACACGTGATTGATCCTACGAAATAGACGGG
27	Km-F	GATCAATCACGTGTGGAATTGTGAGCGGATAACAATTTC
28	km-R	CCGCTTGTCCTTAGAAAAACTCATCGAGCATCAAATGAAAC
29	7002dnaAds-F	GTTTTTCTAAGGACAAGCGGCCCACATC
30	7002dnaAds-R	GGAATGTTTGCCCGGTTTGCC
31	7002dnaAus-F	CTCTTTGAACTGGCGAAAGAACTG
32	7002dnaAus-R	CGAATTCCATCGTGATTGATCCTACGAAATAGACGGG
33	HA-F	ATCAATCACGATGGAATTCGAGCTCCACCGC
34	7002dnaA-R	CACCGCTGCGCTAGAGGTTTTGGTGATGGTGATTAATGC
35	Spec-F	AAACCTCTAGCGCAGCGGTGGTAACGGC
36	Spec-R	CCGCTTGTCCTTATTTGCCGACTACCTTGGTGATCTC
37	7002dnaAds-F	CGGCAAATAAGGACAAGCGGCCCACATCGTAC
38	7002dnaAds-R	GGCCCTGGTCGGTTGAAATAAGC
39	HA-F for confirm	CCCATACGATGTTCCTGACTATGC
40	6803dnaA-us-F	GGGCAAAGTGACAGGATTGGC
41	6803dnaA-us-R	CTCGAATTCCATCAAAAATATACAAATATACTTAGTAGG
42	HA-F for 6803dnaA	GTATATTTTTGATGGAATTCGAGCTCCACCG
43	6803dnaA-R	CACCGCTGCGCTAGGATTCCGGGGCTTGGC
44	spec-F	CCGGAATCCTAGCGCAGCGGTGGTAACGGC
45	spec-R	GATCGCCAGGGGTTTATTTGCCGACTACCTTGGTGATCTC
46	6803dnaA-ds-F	TCGGCAAATAAACCCCTGGCGATCGGC
47	6803dnaA-ds-R	ACCGAAGGCACCGTGGAG
48	a	CGTCATTGATCAATCTCCCATCGGC
49	b	CCTTGCATACCTCACACTGCAC
50	c	GATAACCCAGACTATAGCGACCGG
51	d	GAATTTGAATCGTTTCTTTCATGCAGCC
52	dnaN-target-f	GGGGTCGATCGCTGAAAGTCTTGC
53	dnaN-target-r	GGGGAGGCTGCCCGAGCCTTTG
54	1294-target-f	CAGACGATCGCTATTTAGTCCAG
55	1294-target-r	GGCGGAATTCTGAAACTCTTATC
